# Myrtenol's Effectiveness against Hospital-Acquired Methicillin-Resistant *Staphylococcus aureus*: Targeting Antibiofilm and Antivirulence Properties

**DOI:** 10.1155/2024/8832448

**Published:** 2024-10-16

**Authors:** Amal F. Makled, Azza Z. Labeeb, Eman A. E. Badr, Amany M. Abdelmaksoud, Safa R. Elfiky, Asmaa K. Amer, Asmaa S. Sleem

**Affiliations:** ^1^Department of Medical Microbiology and Immunology, Faculty of Medicine, Menoufa University, Shebin Al Kom, Egypt; ^2^Department of Medical Biochemistry and Molecular Biology, Faculty of Medicine, Menoufa University, Shebin Al Kom, Egypt; ^3^Department of Clinical Pathology, Faculty of Medicine, Menoufa University, Shebin Al Kom, Egypt; ^4^Department of Clinical Pharmacology, Faculty of Medicine, Menoufa University, Shebin Al Kom, Egypt

## Abstract

The emergence of methicillin-resistant *Staphylococcus aureus* (MRSA) several years ago highlighted the challenge of multidrug-resistant infections, emphasizing the critical need for innovative treatment approaches. Myrtenol, known for its antibacterial and antibiofilm properties, holds promise as a potential treatment option. This study aimed to evaluate the effectiveness of myrtenol against MRSA. The collected MRSA isolates were assessed for antimicrobial susceptibility following the Clinical and Laboratory Standards Institute (CLSI) guidelines 2023. Biofilm formation by MRSA was evaluated using the tissue culture plate (TCP) technique. The minimal inhibitory concentration (MIC), minimal bactericidal concentration (MBC), and minimal biofilm inhibitory concentration (MBIC) of myrtenol against MRSA were determined both individually and in combination with antibiotics. Real-time PCR was employed to investigate the impact of myrtenol on the expression of virulence genes (sarA, agrA, and icaD) across the isolates. In this study, MRSA was identified in 90 out of 400 cases (22.5%) of hospital-acquired pathogens. Among the collected MRSA isolates, 53 out of 90 (59%) were found to produce biofilms. The MIC of myrtenol was comparable to the MBC across all tested isolates, they were almost the same. Combinations of myrtenol with most tested antibiotics exhibited synergistic effects exceeding 60%. Among the 53 biofilm-producing isolates, 45 isolates (85%) expressed the sarA gene, 49% expressed the agrA gene, and all biofilm-producing MRSA isolates (100%) expressed the icaD gene. A notable reduction in the relative quantity (RQ) values of virulence gene expression was observed after treatment with the MBIC of myrtenol across all tested isolates. Myrtenol demonstrated strong antimicrobial activity against MRSA, notably reducing the expression of key virulence genes linked to biofilm formation. This suggests its potential as a therapeutic agent for treating biofilm-associated MRSA infections.

## 1. Introduction

Methicillin-resistant *Staphylococcus aureus* (MRSA) was identified by the World Health Organization as one of the multidrug-resistant (MDR) pathogens of great concern [1]. MRSA was also listed in the top of 2019 antibiotic resistance threat report by the Center for Disease Control and Prevention (CDC) [2].

MRSA has now become endemic in many healthcare facilities worldwide, and as a result, it has been the main target of international infection control measures, being responsible for abroad range of infections [3].

MRSA's resistance and chronicity are mainly due to its capability to develop biofilms on both biotic and abiotic surfaces [4, 5]. Biofilm communities of interconnected microorganisms surrounded by an exopolysaccharide (EPS) matrix with proteins and extracellular DNA (eDNA) can foster thousand times more antibiotic resistance in sessile bacteria than in planktonic ones [6].

Biofilm developments are mostly governed by global regulatory systems, including staphylococcal accessory regulator A (*sarA*) and accessory gene regulator (*agr*) [7]. *SarA* modifies the expression of virulence genes by binding to the intergenic region between P2 and P3 promoters of *agr* regulon. Consequently, *sarA* is regarded as a potential target for antimicrobial research since it is a major modulator reducing virulence genes expression as well as biofilm formation [8, 9].

Recently, many studies focused on the exploration of natural antibiofilm compounds against MRSA like; quercetin and tannic acid, stilbenes, alizarin, *α*-mangostin, myrtenol, and nerolidol [10, 11].

Myrtenol has sparked considerable interest due to its pharmacological effects, especially for the remediation of chronic inflammatory disorders. Thus, several researchs were designed to evaluate the impacts of myrtenol, as an herbal extract on resistant bacterial infections [12].

Myrtenol demonstrated a concentration-dependent biofilm inhibitory action. Furthermore, it can prevent essential virulence factors secretion such as lipase, autolysin, slime, and *α*-hemolysin. In brief, myrtenol could be an alternative approach to fight chronic MRSA infections [4, 8].

The objective of this study was to assess hospital-acquired MRSA prevalence and antimicrobial resistance, identify biofilm-producing MRSA strains, determine myrtenol's minimal inhibitory concentration against MRSA, demonstrate myrtenol's efficacy in combating MRSA virulence and biofilm formation, and investigate myrtenol's impact on biofilm-related genes (*sarA*, *agrA*, and, *icaD*) expression using quantitative real-time PCR.

## 2. Patients and Methods

### 2.1. Study Design and Ethical Considerations

This study was carried out at the Department of Medical Microbiology and Immunology, Faculty of Medicine, Menoufia University, Egypt, during the period from January 2022 to June 2023. Inclusion criteria encompassed hospitalized patients across various hospital departments and intensive care units (ICUs) exhibiting hospital-acquired infections that emerged more than 48 hours after admission, while patients who declined participation in the study or those colonized without signs of infection were excluded.

Prior to their participation in the study, informed consent was obtained from each patient. The study protocol was approved by the local Ethics Committee of the Faculty of Medicine, Menoufia University (IRP12/2021MICRO26).

### 2.2. Collection of Clinical Samples and Identification of *Staphylococcus aureus*

Various clinical specimens were obtained from 400 patients who were admitted to different hospital departments and intensive care units (ICUs) of Menoufia university hospitals (MUHs) with variable clinical types of hospital-acquired infections (HAIs). *Staphylococcus aureus* isolates were identified using standard microbiological techniques (microscopic appearance (Gram +ve cocci arranged in grape like clusters.), culture characteristics (mannitol fermenter, beta hemolysis on blood agar), and conventional biochemical reactions in which *S. aureus* is catalase and coagulase positive [13] and confirmed with the Vitek-2 Compact System (bioMerieux, France).

### 2.3. Antimicrobial Susceptibility Testing

The antimicrobial susceptibility of *S. aureus* isolates was performed by the disk diffusion method using various antimicrobial agents (Oxoid, UK) and interpreted in accordance with the CLSI guidelines, 2023. The MIC for vancomycin was determined using the agar dilution method as per CLSI (2023) guidelines. To identify MRSA strains, the cefoxitin (FOX; 30 *μ*g) disk diffusion test was utilized as a proxy, followed by confirmation through minimal inhibitory concentration (MIC) determination using the agar dilution method [14]. For assessing the multiresistance of *S. aureus* isolates, the Multiple Antimicrobial Resistance (MAR) index was calculated using the equation: MAR index = □(a/b) where “a” represents the number of antimicrobials to which the pathogen exhibited resistance, and “b” denotes the total number of antimicrobials tested [15]. The *D*-zone test was performed by disk diffusion method to detect macrolide-inducible resistance to clindamycin according to CLSI (2023) recommendations.

### 2.4. Assessment of MRSA Biofilm Formation and Myrtenol Anti-biofilm Activity

MRSA isolates were further screened for biofilm formation using the tissue culture plate (TCP) method [16]. MRSA strains were cultured overnight in 5 mL of trypticase soy broth with 1% glucose (TSBG), adjusted to 0.5 McFarland standard, and subsequently diluted 1 : 100 in TSBG. Then, 200 *μ*L of each diluted culture was inoculated into triplicate wells of a 96-well plate. After incubation, plates were washed thrice with 200 *μ*L of phosphate-buffered saline (PBS), and adherent bacterial cells were fixed with methanol and stained with crystal violet. Following another round of washing and air-drying, 33% glacial acetic acid was used to dissolve the adherent cells. Optical density (OD) at 590 nm was measured using a plate reader (ELx800, Biotek, USA) to assess biofilm formation intensity. The biofilm's strength was categorized based on standard deviation-calculated OD values:

1. O.D. ≤ O.D.c (O.D. of the negative control) = nonadherent.
2. O.D.c < *O*.D. ≤ (2 × O.D.c) = weakly adherent.
3. (2 × O.D.c) < O.D. ≤ (4 × O.D.c) = moderately adherent.
4. (4 × O.D.c) < O.D. = strongly adherent.

Furthermore, the antibiofilm potential of myrtenol against MRSA was assessed in vitro. The TCP test was conducted with varying concentrations (25, 50, 100, 150, 200, 250, 300, 350, 400, 450, 500, 550, and 600 *μ*g/mL) of myrtenol to investigate its antibiofilm effect. The degree of biofilm inhibition was quantified using the following formula.

(1) $$\begin{array}{c} \left(\%\right)=\left(\frac{\left(\text{Control OD }590\text{nm}-\text{Treated OD }590\text{nm}\right)}{\text{Control OD }590\text{nm}}\right)\times 100\left(8\right). \end{array}$$

### 2.5. Determination of Minimum Inhibitory Concentration (MIC) and Minimum Bactericidal Concentration (MBC) of Myrtenol

The MIC and MBC of myrtenol were determined using the broth microdilution technique in a 96-well plate, following standard procedures [17]. Myrtenol was dissolved in dimethyl sulfoxide (DMSO) (as a solvent only without antibiofim or antimicrobial activity) at varying concentrations (25, 50, 100, 150, 200, 250, 300, 350, 400, 450, 500, 550, and 600 *μ*g/mL). The negative control contained DMSO and broth. This method allows for establishing the minimum concentration of myrtenol needed to inhibit bacterial growth (MIC) and concentration needed to eradicate the bacteria (MBC), providing important insights into myrtenol's antimicrobial effectiveness against the tested bacterial strains.

### 2.6. Assessment of Myrtenol's Synergistic Effect with Tested Antibiotics Using the Checkerboard Method [18]

Serial dilutions of each antibiotic (gentamicin, ciprofloxacin, erythromycin, azithromycin, tetracycline, doxycycline, penicillin, cefoxitin, clindamycin, rifampicin, nitrofurantoin, and trimethoprim sulfamethoxazol) and mytrenol various concentrations (25, 50, 100, 150, 200, 250, 300, 350, 400, 450, 500, 550, and 600 *μ*g/mL) were prepared, incorporating concentrations at least double the previously determined MIC. The negative control contained only DMSO and broth. Antibiotics and myrtenol were assessed both individually and in combination. The combination effect of myrtenol with antibacterial drugs was evaluated using the fractional inhibitory concentration index (FICI). The FICI was calculated using the fractional inhibitory concentrations (FIC) of the substances involved as follows:

(2) $$\begin{array}{c} \text{FICI}=\text{FICA}+\text{FICB}, \end{array}$$

where

(3) $$\begin{array}{c} \text{FICA}=\left(\frac{\text{MIC of substance }A\text{ in combination}}{\text{MIC of substance }A\text{ alone}}\right)\text{ and},\\ \text{FICB}=\left(\frac{\text{MIC of substance }B\text{ in combination}}{\text{MIC of substance }B\text{ alone}}\right). \end{array}$$

### 2.7. Quantification of Extracellular DNA (eDNA) in MRSA Biofilms Using Agarose Gel Electrophoresis

The quantity of extracellular DNA (eDNA) within MRSA biofilms was determined using agarose gel electrophoresis. MRSA was allowed to form biofilm in the presence or absence of varying concentrations of myrtenol, with removal of planktonic cells. The biofilm-containing wells were treated with 1 mL of TE buffer (10 mM Tris, 1 mM EDTA [pH 8]) and incubated at 65°C for 15 minutes. Adherent cells were then scraped off, followed by centrifugation at 13,000 rpm for ten minutes to pellet the cells. The resulting cell pellets were resuspended in 200 *µ*L of TE buffer and centrifuged again at 8000 rpm for 10 minutes. The supernatant containing eDNA was collected and subjected to visualization using 1.5% (w/v) agarose gel electrophoresis, as described by Kaplan et al. [19] and Selvaraj et al. [4].

### 2.8. Genotypic Detection of *sarA*, *agrA*, and *icaD* Genes in MRSA Isolates

RNA extraction and purification were performed on MRSA isolates, including 53 biofilm-producing and 37 nonproducing isolates, using the GeneJET™ RNA Purification Kit (Thermo Fisher Scientific, UK). Total RNA was then reverse transcribed into complementary DNA (cDNA) using a cDNA kit from NORGEN BIOTEK (Canada). The primer sequences used for detecting *sarA*, *agrA*, and *icaD* genes were obtained from Selvaraj et al., 2019 [8], and are detailed in Table 1, sourced from Invitrogen (Thermo Fisher, UK). The gyrB gene was used as positive control.

After identifying biofilm-producing MRSA isolates expressing *sarA*, *icaD*, and *agrA* genes, these isolates were retested for gene expression following treatment with the minimal biofilm inhibitory concentration of myrtenol.

Relative quantification (RQ) determined the change in expression of the nucleic acid sequence in the test sample (target) relative to the same sequence in the control sample. The expression of study genes was calculated using the comparative ∆∆Ct method, where the amount of specific gene expression was adjusted to GyRB expression and compared to a reference control. The comparative ∆∆Ct method was calculated using the following equation: normalized target gene expression (Fold change) = (2)^ (-∆∆Ct) folds. In this equation, ∆∆Ct = ∆Ct patient sample–∆Ct control sample. Here, ∆Ct sample represented the Ct value for the target gene normalized to the Ct value for the endogenous housekeeping gene, expressed as fold changes.

### 2.9. Statistical Analysis

The Statistical Package for the Social Sciences (SPSS) version 29 software was employed for data analysis. The significance of qualitative data was assessed using the Chi-square test. To compare variances among two or more groups and determine significant differences, the F-test and post hoc tests were utilized. A significance level of *p*  <  0.05 was considered statistically significant.

## 3. Results

This study analyzed 120 *Staphylococcus aureus* isolates obtained from various clinical specimens, of which 90/120 were identified as MRSA (75%). The MRSA isolates demonstrated significantly high antimicrobial resistance rates, i.e., 73% to gentamicin, 71% to ciprofloxacin and 70% to rifampicin. Notably, all MRSA and MSSA isolates were completely sensitive (100%) to both linezolid and vancomycin (Table 2, supplementary tables 1 and 2).

In the assessment of biofilm formation using the MTP method (supplementary table 3), 59% (53/90) of MRSA isolates were identified as biofilm producers, categorized as 32.1% weak, 49% moderate, and 18.9% strong producers. The resistance rates for biofilm producers were markedly higher compared to nonbiofilm producers: rifampicin (87% vs. 46%), gentamicin (87% vs. 54%), ciprofloxacin (81% vs. 57%), erythromycin (75% vs. 46%), tetracycline (74% vs. 43%), nitrofurantoin (70% vs. 41%), clindamycin (68% vs. 32%), azithromycin (66% vs. 57%), and doxycycline (64% vs. 27%). All observed differences were statistically significant (*p*  <  0.001).

In this study, the antibacterial efficacy of myrtenol was investigated. The MIC of myrtenol was found to be equivalent to its MBC for all tested MRSA isolates. The MIC50 of myrtenol, representing the concentration at which 50% of biofilm-producing MRSA isolates were inhibited, was determined to be 250 *µ*g/ml. Additionally, the MIC90, indicating the concentration at which 90% of the isolates were inhibited, was 450 *µ*g/ml (Table 3).

Using the checkerboard method to evaluate the combinatory effects of myrtenol with various antibiotics, our data demonstrated synergism with gentamicin (77.4%), ciprofloxacin (69.8%), erythromycin (86.8%), azithromycin (83.0%), tetracycline (79.2%), doxycycline (88.7%), penicillin (66.0%), cefoxitin (67.9%), clindamycin (81.1%), rifampicin (64.2%), nitrofurantoin (60.4%), and trimethoprim-sulfamethoxazole (77.4%). All showed a fractional inhibitory concentration index (FICI) of less than 0.5 (Table 4).

**Figure 1:**
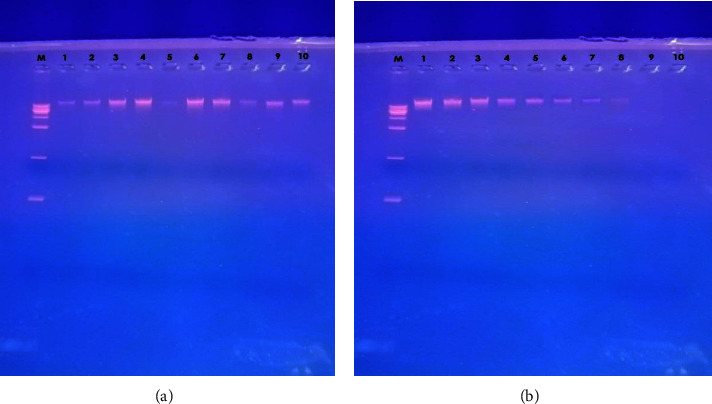
Detection of extracellular DNA (eDNA) in MRSA Biofilm with and without myrtenol treatment. (a) M: 1 K ladder, Lane 4, 6, 7: eDNA from strong biofilm producers, Lane 3, 9, 10: eDNA from moderate biofilm producers, Lane 1, 2, 5, 8: eDNA from weak biofilm producers, (b) M: 1 K ladder, Lane 1: eDNA from strong biofilm-producing MRSA without myrtenol, Lane 2–10: Extracellular DNA detected under the effect of increasing concentrations of myrtenol (25, 50, 100, 150, 200, 250, 300, 350, 400, and 450 *µ*g/ml).

Extracellular DNA extracted from all biofilm-producing MRSA isolates was notably decreased by varying concentrations of myrtenol (Figure 1).

**Figure 2:**
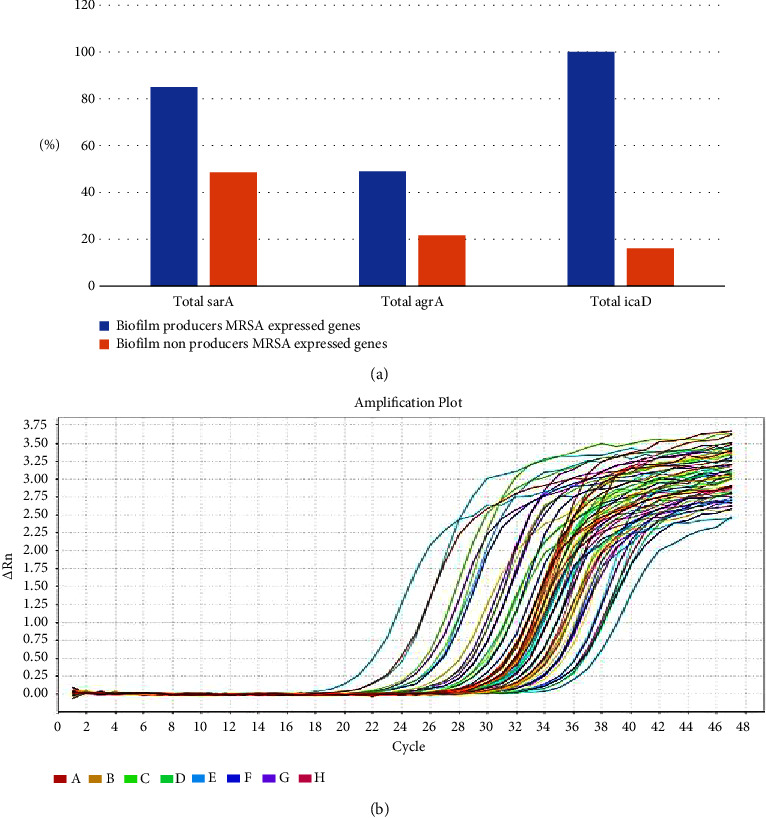
Gene expression analysis of *icaD*, *sarA*, and *agrA* in biofilm-producing MRSA isolates using real-time PCR. (a) Expression of *icaD*, *sarA*, and *agrA* genes among biofilm-producing MRSA isolates detected by real-time PCR and (b) amplification plot curve of expressed virulence genes.

In our investigation of gene expression levels *(icaD*, *sarA*, and *agrA*) among MRSA strains using real-time PCR (Figure 2), we found a positive correlation between these gene expressions and biofilm production in MRSA strains (supplementary table 4). Biofilm-producing MRSA isolates exhibited significantly higher expression levels of *sarA*, *agrA*, and *icaD* genes compared to nonbiofilm-producing MRSA strains, with statistical significance (*p*  <  0.05). Among the 53 biofilm-producing MRSA isolates, 45 strains (85%) expressed the *sarA* gene, 26 strains (49%) expressed the *agrA* gene, and all biofilm-producing isolates (100%) expressed the *icaD* gene. In contrast, among the nonbiofilm producers, only 18 strains (48.6%) expressed the *sarA* gene, 8 strains (21.6%) expressed the *agrA* gene, and 6 strains (16.2%) expressed the *icaD* gene.

**Figure 3:**
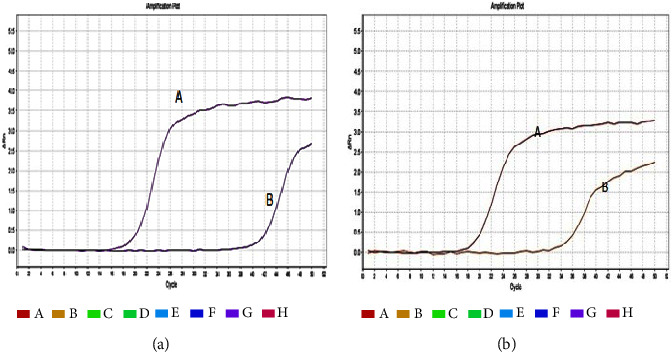
Comparison of amplification plots pre- (a) and posttreatment (b) with myrtenol.

A notable reduction in the relative quantity (RQ) values of the expressed genes *sarA*, *agrA*, and *icaD* were observed after treatment with the MBIC of myrtenol across all strong, moderate, and weak biofilm-producing strains. These changes were statistically significant (*p*  <  0.05), as shown in Table 5 and Figure 3.

## 4. Discussion

Methicillin-resistant *Staphylococcus aureus* (MRSA) poses a significant threat to public health worldwide. One of the primary drivers of its antimicrobial resistance is the formation of biofilms. Consequently, inhibiting biofilm formation has become a promising strategy for managing resistant infections [4]. Our study focused on the antivirulence and antibiofilm properties of myrtenol against clinical isolates of MRSA that produce biofilms.

Out of 120 *Staphylococcus aureus* isolates, 75% were identified as MRSA, consistent with findings from various Arabian and European countries [7, 15, 20–22]. However, different prevalence rates were observed in Egypt (94%) [23], Nepal (26.4%) [24], and China (17%) [25]. These variations may stem from differences in circulating clones, infection control protocols, and antibiotic prescription patterns in different healthcare settings.

MRSA is a major contributor to antibiotic-resistant infections globally [15, 22–25]. Our study confirmed this, as all MRSA isolates exhibited high resistance to most tested antibiotics. On a positive note, all MRSA isolates were sensitive to linezolid and vancomycin. Similar results were reported in previous Egyptian studies [23, 26, 27] where MRSA isolates showed high resistance to various antibiotics, except vancomycin. The Multiple Antibiotic Resistance (MAR) index revealed that 77.5% of isolates had a MAR index of ≥0.2, indicating a high rate of antibiotic use in Egypt, consistent with findings by Sonbol et al. [15].

MRSA's capacity to form biofilms, leading to persistent infections, placed it high on the priority list of health organizations [17]. In the current study, the microtiter plate method detected biofilm production in 59% of MRSA isolates. Our findings aligned with those reported by Mathur et al. [28] in India and Samadi et al. [29] in Iran. Conversely, studies by Derakhshan et al. [30] in Iran and Abdrabaa et al. [31] in Iraq reported that 100% of MRSA isolates were biofilm producers. These discrepancies may stem from the use of different biofilm detection methods. As biofilm inhibition could be a promising alternative therapy for MRSA persistent infections [4], we examined the minimal inhibitory concentration (MIC) of myrtenol against biofilm-producing isolates. Our results indicated that the MIC was equal to the minimal bactericidal concentration (MBC) for all tested isolates; 200 *µ*g/mL was the most common MIC among isolates by 22.6%. This is consistent with the findings of Cordeiro et al. [8], who reported both MIC and MBC values of 128 *µ*g/mL for all *S. aureus* strains. Similarly, Mahmoud et al. [32] observed equal MIC and MBC values for myrtenol against their staphylococcal isolates. However, Selvaraj et al. [4] identified a higher MIC of 600 *µ*g/mL for myrtenol against MRSA. The antibacterial activity of myrtenol against various Gram-negative organisms was also studied by Al-Mariri et al. [33], with MIC values ranging from 25 to 50 *µ*L/mL. These variations in MIC values can be attributed to differences in bacterial strains and the number of isolates used in different studies [8]. In addition, myrtenol exhibited significant biofilm inhibition against MRSA at all tested concentrations, with more than 90% inhibition observed from the minimum inhibitory concentration (MIC) up to 600 *µ*g/mL. Several studies have corroborated our findings on the antibiofilm activity of myrtenol [4, 8].

Extracellular DNA (eDNA) is widely distributed in *S. aureus* biofilms and is believed to play a crucial role in antibiotic resistance development, horizontal gene transfer, and biofilm stability enhancement^19^. In our study, we observed a decrease in eDNA release in myrtenol-treated cells. Similar results were reported by Selvaraj et al. [4] and Cordeiro et al. [8], who found that myrtenol significantly limited eDNA release in their samples.

Our research demonstrated a synergistic effect between myrtenol and various antibiotics, enhancing their efficacy by more than 60% and up to 88.7%. This was reflected in a fractional inhibitory concentration index (FICI) of less than 0.5, consistent with the findings of Mahmoud et al. [32] and Cordeiro et al. [8]. The combination of natural compounds and antimicrobials had proven to be highly effective, offering promising potential for clinical application against resistant organisms [8]. Moreover, the required doses of each medication and their dose-dependent toxic effects can be significantly reduced when these substances are used in combination without affecting their pharmacokinetics.

In the current study, 85%, 49%, and 100% of biofilm-producing MRSA isolates expressed the *sarA*, *agrA*, and *icaD* genes, respectively. In contrast, nonbiofilm producers expressed these genes at rates of 48.6%, 21.6%, and 16.2%. These findings coincided with the results of Azmi et al. [34] in Palestine, who reported *sarA* in 69.8%, *agrA* in 39.5%, and *icaD* in 83.5% of MRSA isolates. In addition, a study by Mashaly and Badr [35] in Egypt found the *icaD* gene in 90.6% of MRSA isolates and in all biofilm-forming isolates. Similarly, Rezk et al. [36] detected the *icaD* gene in all isolates and the *agrA* gene in 54% of isolates. The *icaD* gene cluster encodes polysaccharide intercellular adhesion proteins, which are crucial for biofilm formation in *Staphylococcus aureus*. However, negative translational or posttranslational regulation, or point mutations in this gene, can impact biofilm production and strength [35–37].

We observed a marked decrease in RQ values of the expressed genes *sarA*, *agrA*, and *icaD* after treatment with the minimal biofilm inhibitory concentration (MBIC) of myrtenol across all biofilm-producing isolates, consistent with several previous reports [4, 8, 38, 39]. Important virulence genes are regulated by *SarA*, either dependently or independently of the agr regulatory system. The agr regulatory system's promoter region was bounded by *SarA*, which increases the expression of these virulence genes. Thus, the reduction of *sarA* and *agrA* expression in the presence of myrtenol significantly reduces MRSA pathogenicity, highlighting the encouraging antimicrobial efficacy of myrtenol [4].

## 5. Conclusions

Our study underscored the significant bactericidal and antibiofilm properties of myrtenol against MRSA. The synergistic effects observed when myrtenol was combined with antibacterial agents suggest its potential for effective use in vitro. Notably, myrtenol demonstrated potent antibiofilm activity even at subinhibitory concentrations, highlighting its ability to inhibit biofilm formation. These findings highlight myrtenol as a promising therapeutic agent for combating drug-resistant MRSA infections, either as a standalone treatment or in combination with other medications. Further research is warranted to explore its clinical applications and potential benefits in treating MRSA infections both in vivo and in vitro.

## Figures and Tables

**Table 1:** Primer sequences for genes of interest.

Genes	Forward primer	Reverse
*gyrB*	5′-GGTGCTGGGCAAATACAAGT-3′	5′-TCCCACACTAAATGGTGCAA-3′
*sarA*	5′-CAAACAACCACAAGTTGTTAAAGC-3′	5′-TGTTTGCTTCAGTGATTCGTTT-3′
*icaD*	5′-ATGGTCAAGCCCAGACAGAG-3′	5′-AGTATTTTCAATGTTTAAAGCA-3′
*agrA*	5′-TGATAATCCTTATGAGGTGCTT-3′	5′-CACTGTGACTCGTAACGAAAA-3′

This table should be involved in material and methods under the paragraph. The primer sequences used for detecting *sarA*, *agrA* and *IcaD* genes were obtained from Selvaraj et al., 2019.

**Table 2:** Antibiogram of *Staphylococcus aureus* isolates (*n* = 120) based on methicillin resistance.

Antimicrobial groups	Antimicrobial agent	Abbrev	Disk content	MRSA (*n* = 90)						MSSA (*n* = 30)						X^2^	*p* value
				S		I		R		S		I		R			
				NO	%	NO	%	NO	%	NO	%	NO	%	NO	%		
Macrolids	Azithromycin	AZM	15 *μ*g	22	24	12	13	56	62	12	40	3	10	15	50	2.69	0.26
	Erythromycin	E	15 *μ*g	21	23	12	13	57	63	16	53	2	7	12	40	9.55	0.008

Tetracyclines	Doxycycline	DO	30 *μ*g	28	31	18	20	44	49	11	37	5	17	14	47	0.36	0.83
	Tetracycline	TE	30 *μ*g	25	28	10	11	55	61	10	33	7	23	13	43	3.87	0.14

Ansamycins	Rifampin	RA	5 *μ*g	24	27	3	3	63	70	13	43	2	7	15	50	4	0.13

Nitrofurans	Nitrofurantoin	F	300 *μ*g	23	26	15	17	52	58	14	47	5	17	11	37	5.16	0.07

Aminoglycosides	Gentamicin	CN	10 *μ*g	18	20	6	7	66	73	14	47	6	20	10	33	15.7	0.0004

Oxazolidinones	Linezolid	LZD	30 *μ*g	87	96.7	3	3.3	0	0	30	100	0	0	0	0	NA	NA

Fluoroquinolones	Ciprofloxacin	CIP	5 *μ*g	16	18	10	11	64	71	16	53	6	20	8	27	19.4	<0.0001

Folate pathway inhibitors	Sulfamethoxazol trimethoprim	SXT	1.25/23.75 *μ*g	37	41	8	9	45	50	15	50	4	13	11	37	1.7	0.42

Clindamycins	Clindamycin	DA	2 *μ*g	36	40	6	7	48	53	20	67	2	7	8	27	6.86	0.03

Glycopeptides	Vancomycin (MIC)	VA	—	86	95.6	4	4.4	0	0	30	100	0	0	0	0	NA	NA

*β*-lactams	Cefoxitin (MIC)	FOX	—	0	0	0	0	90	100	30	100	0	0	0	0	N/A	N/A
	Penicillin	P	10 units	0	0	0	0	90	100	6	20	0	0	24	80	14.6	0.0001

S, susceptible I, intermediate *R*, resistant, X2: Chi-square test. MRSA: methicillin-resistant *Staphylococcus aureus*, MSSA: methicillin-susceptible *Staphylococcus aureus*

**Table 3:** Determination of MIC, MBC, and MBIC of myrtenol against biofilm-producing MRSA.

Myrtenol concentrations	Biofilm producers (*n* = 53)			
	Minimal inhibitory concentration (MIC = MBC)		Minimal biofilm inhibitory concentration (MBIC)	
	No	%	No	%
25 *µ*g/ml	0	0%	1	1.9%
50 *µ*g/ml	0	0%	5	9.4%
100 *µ*g/ml	2	3.8%	15	28.3%
150 *µ*g/ml	10	18.9	9	17%
200 *µ*g/ml	12	22.6%	10	18.9%
250 *µ*g/ml	8	15%	8	15%
300 *µ*g/ml	6	11.3%	3	5.7%
350 *µ*g/ml	5	9.4%	1	1.9%
400 *µ*g/ml	3	5.7%	1	1.9%
450 *µ*g/ml	3	5.7%	0	0%
500 *µ*g/ml	2	3.8%	0	0%
550 *µ*g/ml	1	1.9%	0	0%
600 *µ*g/ml	1	1.9%	0	0%
MIC_50_	250 *µ*g/ml			
MIC_90_	450 *µ*g/ml			

MIC: Minimal inhibitory concentration, MBC: Minimal bactericidal concentration, MBIC: Minimal biofilm inhibitory concentration, MIC_50_: MIC of myrtenol at which 50% of the isolates were inhibited. MIC_90_: MIC of myrtenol at which 90% of the isolates were inhibited.

**Table 4:** Evaluation of myrtenol-antibiotic interaction in biofilm-producing MRSA isolates via the checkerboard method.

Effect	Number of isolates		FICI	Combination
	%	No		
Synergism	77.4%	41	<0.5	Myrtenol and gentamicin
Additivity	22.6%	12	>0.5 < 1	
Synergism	69.8%	37	<0.5	Myrtenol and ciprofloxacin
Additivity	30.2%	16	>0.5 < 1	
Synergism	86.8%	46	<0.5	Myrtenol and erythromycin
Additivity	13.2%	7	>0.5 < 1	
Synergism	83.0%	44	<0.5	Myrtenol and azithromycin
Additivity	17.0%	9	> 0.5 < 1	
Synergism	79.2%	42	<0.5	Myrtenol and tetracycline
Additivity	15.1%	8	>0.5 < 1	
Indifference	5.7%	3	≥1 < 4	
Synergism	88.7%	47	<0.5	Myrtenol and doxycycline
Additivity	11.3%	6	>0.5 < 1	
Synergism	66.0%	35	<0.5	Myrtenol and penicillin
Additivity	17.0%	9	>0.5 < 1	
Indifference	17.0%	9	≥1 <4	
Synergism	67.9%	36	<0.5	Myrtenol and cefoxitin
Additivity	9.4%	5	>0.5 < 1	
Indifference	22.6%	12	≥1 < 4	
Synergism	81.1%	43	<0.5	Myrtenol and clindamycin
Additivity	15.1%	8	>0.5 < 1	
Indifference	3.8%	2	≥1 < 4	
Synergism	64.2%	34	<0.5	Myrtenol and rifampicin
Additivity	11.3%	6	>0.5 < 1	
Indifference	24.5%	13	≥1 < 4	
Synergism	60.4%	32	<0.5	Myrtenol and nitrofurantoin
Additivity	17	9	>0.5 < 1	
Indifference	22.6%	12	≥1 < 4	
Synergism	77.4%	41	<0.5	Myrtenol and trimethoprim sulfamethoxazol
Additivity	9.4%	5	>0.5 < 1	
Indifference	13.2%	7	≥1 < 4	

FICI: fractional inhibitory concentration index. synergism: FICI <0.5, additivity: FICI >0.5–1, indifference: FICI >1–2 and antagonism: FICI ≥2.

**Table 5:** Relative quantification (RQ) of gene expression before and after MRSA treatment with MBIC of myrtenol using real-time PCR.

Genes	RQ of expressed genes		Biofilm producing MRSA			*F*-Test	*p* value	Post hoc test		
			Week	Moderate	Strong			I^∗^	II^∗^	III^∗^
*agrA*	Before	Mean	0.331	3.604	7.697	4.318	0.048	<0.001	<0.001	<0.001
		SD	0.109	1.024	2.899					
	After	Mean	0.011	0.562	4.850					
		SD	0.009	0.132	0.722					

*IcaD*	Before	Mean	75.205	202.205	500.794	4.227	0.051	<0.001	<0.001	<0.001
		SD	20.436	60.272	152.447					
	After	Mean	10.006	90.486	280.954					
		SD	2.228	22.621	93.605					

*SarA*	Before	Mean	1.371	9.555	20.557	4.256	0.056	<0.001	<0.001	<0.001
		SD	0.451	2.851	6.743					
	After	Mean	0.094	2.115	9.953					
		SD	0.004	0.796	3.293					

I^∗^: weak versus moderate biofilm producers. II^∗^: moderate versus strong biofilm producers. III^∗^: week versus strong biofilm producers.

## Data Availability

The data used to support the findings of this study are available from the corresponding author upon reasonable request.

## Abbreviations

MRSA: Methicillin-resistant *Staphylococcus aureus*
CLSI: Clinical and laboratory standards institute
TCP: Tissue culture plate
MIC: Minimum inhibitory concentration
MBC: Minimum bactericidal concentration
MBIC: Minimal biofilm inhibitory concentration
RQ: Relative quantity
MDR: Multidrug resistant
CDC: Center for disease control and prevention
eDNA: Extracellular DNA
EPS: Exopolysaccharide
sarA: Staphylococcal accessory regulator A
agr: Accessory gene regulator.

## Additional Points

*Graphical abstract*. Illustrates summary of our study.

## Supplementary Materials

Supplementary Materials Supplementary Table (1): antimicrobial resistance patterns and multiple antimicrobial resistance (MAR) index among *Staphylococcus aureus* isolates (*n* = 120). Supplementary Table (2): phenotyping results of MRSA/MSSA species and inducible clindamycin resistance. Supplementary Table (3): detection of biofilm production among MRSA isolates using the tube method and microtitre plate (MTP) method. Supplementary Table (4): RQ of different expressed genes by real-time PCR.
