# Supplementary material for: Myrtenol's Effectiveness against Hospital-Acquired Methicillin-Resistant Staphylococcus aureus: Targeting Antibiofilm and Antivirulence Properties
Source: Int J Microbiol. 2024 Oct 16;2024:8832448. doi: 10.1155/2024/8832448 (PMC11498983; doi:10.1155/2024/8832448)
Supplement: Supplementary Materials — Supplementary Table (1): antimicrobial resistance patterns and multiple antimicrobial resistance (MAR) index among Staphylococcus aureus isolates (n = 120). Supplementary Table (2): phenotyping results of MRSA/MSSA species and inducible clindamycin resistance. Supplementary Table (3): detection of biofilm production among MRSA isolates using the tube method and microtitre plate (MTP) method. Supplementary Table (4): RQ of different expressed genes by real-time PCR. [file 8832448.f1.docx]

**Supplementary tables**.

**Supplementary Table (1) Antimicrobial resistance patterns and Multiple antimicrobial resistance (MAR) index among *Staphylococcus. aureus* isolates (n=120).**

| **Character of resistant strain** | **Percentage**  **(%)** | **Isolates number** | **(MAR index)** | **Antimicrobial resistance pattern** | **Code** |
| --- | --- | --- | --- | --- | --- |
| **R** | **18.33%** | **22** | **0.14** | **P-FOX**  **AZM-RD**  **P- Do**  **Do-TE**  **P-E**  **P-TE**  **P-CN**  **P-F** | **SII** |
| **R** | **5%** | **6** | **0.21** | **P-AZ-M-E**  **P-Fox-CN** | **SIII** |
| **R**  **MDR** | **4.17%** | **5** | **0.28** | **P-FOX-E-AZM**  **P-FOX-F-CN**  **P-FOX- CIP –RD** | **SIV** |
| **MDR** | **3.33%** | **4** | **0.42** | **P-FOX-CN-CIP-TE-RD**  **P-FOX-CN- CIP -E-RD** | **SVI** |
| **MDR** | **5%** | **6** | **0. 5** | **P-FOX-CIP-E-AZM-TE-DA**  **P-FOX-CIP-E-DA-RD-F**  **P-FOX-CN-E-DA-AZM-RD** | **SVII** |
| **MDR** | **15.83%** | **19** | **0.57** | **P-FOX-CN-CIP-E-TE-AZM-F**  **P-FOX-CN-CIP-E-AZM-DO-F**  **P-FOX-CN-CIP-E-DA-RD-F**  **P-FOX-CN-E-SXT-AZM-RD-Do**  **P-FOX-E-DA-AZM-CN-RD-SXT**  **P-FOX-E-CN-DA-AZM-TE-RD**  **P-FOX-TE-CN-AZM-E-F-RD** | **SVIII** |
| **MDR** | **23.33%** | **28** | **0.64** | **P-RD-E-AZM-RD-Do-TE-F-SXT**  **P-FOX-CN- CIP -TE-AZM-RD-Do-F**  **P-FOX-CN-AZM-F-DA-CIP-RD-SXT**  **P-FOX-CIP-TE-DA-AZM-RD-DO-F**  **P-FOX-TE-DA-SXT-CIP-RD-F-Do**  **P-FOX-DO-CN-E-SXT-AZM-RD-TE**  **P-FOX-TE-CN-E-DA-CIP-RD- SXT**  **P-FOX-TE-DA-SXT-CIP-RD-Do-F**  **P- Do-E-RD-TE-CIP-SXT-DA-AZM**  **P-FOX-CN- CIP -E-TE-AZM-RD-F** | **SIX** |
| **MDR** | **16.67%** | **20** | **0.71** | **P-TE-E-AZM-DA-DO-F-CN-CIP-SXT**  **P-SXT-AZM-E-RD-DO-F-CN-CIP-DA**  **P-E-AZM-RD-DO-F-CN-CIP-SXT-DA**  **P-FOX-E-TE-AZM-DA-CIP-SXT-Do-F**  **P-FOX-DO-CN-E-DA-SXT-CIP-RD-AZM**  **P-FOX-TE-CN-Do-CIP-F-DA-RD-SXT**  **P-FOX-CN-E-TE-CIP-F-Do-RD-DA**  **P-FOX-DO -CN-E-CIP-F-SXT-AZM-RD** | **SX** |
|  |  |  |  |  |  |
| **XDR** | **6.67%** | **8** | **0.785** | **P-FOX-CN-CIP-E-TE-AZM-DA-RD-Do-F**  **P-E-AZM-SXT-DA-RD-DO-TE-F-CN-CIP**  **P-FOX- E-TE-CIP-F-CN-DO-DA-SXT-RD** | **SXI** |
| **XDR** | **1.67%** | **2** | **0.857** | **P-FOX- -E-TE-CIP-F-DO-DA-SXT-RD- CN-AZM** | **SXII** |

P; Penicillin, FOX; Cefoxitin, VA; Vancomycin, CN; Gentamicin, AZM; Azithromycin, E; Erythromycin, TE; Tetracycline, CIP; Ciprofloxacin, DO; Doxycyclin, DA; Clindamycin, SXT; Trimethoprim-sulfamethoxazole, F; Nitrofurantoin, RD; Rifampin, LZD; Linezolid. MDR (Multi-Drug Resistant). XDR (Extensively Drug-Resistant).

**Supplementary Table (2): Phenotyping Results of MRSA */* MSSA species and Inducible Clindamycin Resistance.**

| **Phenotypes** | **Erythromycin**  **susceptibility**  **result** | **Clindamycin**  **susceptibility**  **result** | **MRSA (n=90)** | | **MSSA (n=30)** | | **X^2^** | **P value** |
| --- | --- | --- | --- | --- | --- | --- | --- | --- |
|  |  |  | **No** | **%** | **No** | **%** |  |  |
| **^a^cMLSB** | Resistant | Resistant | 54 | 60.0% | 10 | 33.3% | 14 | .007 |
| **^b^iMLSB** | Resistant | Susceptible  D test positive | 8 | 8.9% | 2 | 6.7% |  |  |
| **^c^MS** | Resistant | Susceptible  D test negative | 15 | 16.7% | 4 | 13.3% |  |  |
| dL | Susceptible | Resistant | 1 | 1.11% | 2 | 6.7% |  |  |
| eS | Susceptible | Susceptible | 12 | 13.33% | 12 | 40% |  |  |

**MLSB: macrolide lincosamide-streptogramin B family of antibiotics**

**a- cMLSB: constitutive resistance to MLSB antibiotics**

**b- iMLSB: inducible resistance to MLSB antibiotics**

**c- MSB: macrolide-streptogramin B phenotype (resistance only to erythromycin)**

**d- L: resistance only to clindamycin**

**e- S: susceptible to both erythromycin and clindamycin**

**Supplementary Table 3: detection of biofilm production among MRSA isolates using the Tube method and Microtitre plate (MTP) method.**

| **P value** | **X2** | **Total** | **Biofilm non - producers** | | **Biofilm producers** | | **Biofilm detection methods** | |
| --- | --- | --- | --- | --- | --- | --- | --- | --- |
|  |  |  | **%** | **No** | **%** | **No** |  |  |
| **0.23** | **1.43** | **90 (100%)** | **50%** | **45** | **50%** | **45** | **Tube method** | |
|  |  | **90 (100%)** | **41%** | **37** | **59%** | **53** | **Total** | **MTP method** |
|  |  |  |  |  | **32.1%** | **17** | **week biofilm producers** |  |
|  |  |  |  |  | **49%** | **26** | **moderate biofilm producers** |  |
|  |  |  |  |  | **18.9%** | **10** | **strong biofilm producers** |  |

**Supplementary Table (4): RQ of different expressed genes by real time PCR**

|  |  | **RQ of expressed *agrA* gene** | **RQ of expressed *icaD* gene** | **RQ of expressed *sarA* gene** |
| --- | --- | --- | --- | --- |
| **Biofilm forming isolates** | **Mean** | **3.877** | **259.4** | **10.49** |
|  | **± SD** | **1.34** | **77.718** | **3.348** |
|  | **Median** | **3.2157** | **284.257** | **10.489** |
|  | **Range Min –Max** | **0.103-8.932** | **22.8-775.765** | **0.274-29.233** |
| **Control** | **Mean** | **0.624** | **0.664** | **0.565** |
|  | **± SD** | **0.294** | **0.280** | **0.238** |
|  | **Median** | **0.561** | **0.612** | **0.521** |
|  | **Range Min –Max** | **0.26156165-1.202** | **0.3002556-192** | **0.2555674-1.015** |
